# The frequency of *BRCA1* founder mutation c.5266dupC (5382insC) in breast cancer patients from Ukraine

**DOI:** 10.1186/s13053-015-0040-3

**Published:** 2015-10-13

**Authors:** Ielizaveta Gorodetska, Svitlana Serga, Natalia Levkovich, Tetiana Lahuta, Ludmila Ostapchenko, Serhyi Demydov, Nikolay Anikusko, Valeriy Cheshuk, Ivan Smolanka, Svitlana Sklyar, Serhyi Polenkov, Oleksander Boichenko, Iryna Kozeretska

**Affiliations:** 1 Department General and Molecular Genetics, Educational and Scientific Centre “Institute of Biology”, Taras Shevchenko National University of Kyiv, Kyiv, 01601 Ukraine; 2 Shupik National Postgraduate Education Medical Academy, Kyiv, Ukraine; 3 Kyiv Municipal Clinical Oncological Center, Kyiv, Ukraine; 4 Ukrainian National Cancer Institute, Kyiv, Ukraine; 5 Chernihiv Regional Oncology Center, Chernihiv, Ukraine; 6 Zhytomyr Regional Oncology Center, Zhytomyr, Ukraine

**Keywords:** Breast cancer, *BRCA1*, 5382insC, Mutation, Ukrainian population

## Abstract

Germ-line mutations in several genes, such as *BRCA1* and *BRCA2*, are known to increase the risk of breast cancer. These heritable mutations are unequally represented among populations with different ethnic background due to founder effects and thereby contribute to differences in breast cancer rates in different populations. The *BRCA1* mutation c.5266dupC (also known as 5382insC or 5385insC) was detected in a sample of 193 breast cancer patients in Ukraine by multiplex mutagenically separated PCR using published specific primers. Nine *BRCA1* mutations 5382insC were detected (4.7 %). The difference in age of diagnosis (35 years in 5382insC carriers versus 45 years in non-carriers) we observed is consistent with other reports indicating that the 5382insC mutation is a factor of genetic predisposition to breast cancer, which is consistent with reports from other countries.

## Findings

The *BRCA1* gene is critical for a number of important cellular processes, such as maintenance of genome integrity, repair of DNA double-strand breaks, and cell cycle control [1]. Mutations in *BRCA1* and *BRCA2* are associated with an increased risk of breast cancer, and is reported to be as high as 80 % [2], and also associated with ovarian, prostate, pancreatic and male breast cancer [3–5]. Breast cancer is the most frequent type of cancer in Ukrainian women and is the primary cause of cancer-related deaths. A total of 17,537 patients were diagnosed with breast cancer in 2011 (including 130 men) corresponding to a standardized incidence rate of 23.9 per 100,000 individuals [6], similar to that of other Eastern European countries (22.07 in Moldova, 45.86 in Belarus, 50.04 in Romania, 50.54 in Hungary, 51.89 in Poland) but far less than that of the United States (92.93) [7].

Knowledge of the presence of a *BRCA1* mutation is important for both prevention of cancer and personalized treatment. Intensive screening of mutation carriers with magnetic resonance imaging is now recommended in developed countries [8–11]. Genetic testing at the time of diagnosis facilitates choice of treatment and *BRCA1* carriers with breast cancer may benefit from bilateral mastectomy, from oophorectomy and from cisplatinum treatments [12, 13]. Unfortunately, in Ukraine, genetic screening is not currently offered at the time of breast cancer diagnosis [14–18].

The frequency of *BRCA1* mutations has been shown to differ among ethnic backgrounds [13, 19–22], reaching as high as 2.5 % in Ashkenazi Jews [19, 21, 22]. A small number of studies of Ukrainian women breast cancer patients [14–18] have revealed the presence of a founder mutation in *BRCA1,* 5382insC. This is the most common mutation among Slavic patients with breast or ovarian cancer and has been studied extensively in Poland, Russia, Belarus and the Baltic countries. Here we present the results of a screening for the frequency of the *BRCA1* mutations 5382insC among 193 Ukrainian breast cancer patients.

### Patient samples

We screened 193 breast cancer patients diagnosed at different ages. Data on each patient were collected from their clinical records, accessed with the patients’ permission. We documented family history (FH) of disease in 135 patients out of 193 persons. Clinical material was collected at the Shupik National Postgraduate Education Medical Academy, the Kyiv Municipal Clinical Oncological Center, the Ukrainian National Cancer Institute, the Chernihiv Regional Oncology Center, and the Zhytomyr Regional Oncology Center with informed consent and approval from the local ethics committee (committee on Bioethics: order number №16 Educational and Scientific Centre “Institute of Biology”, Taras Shevchenko National University of Kyiv of February 25, 2014, 64 Volodymyrska St., 01601, Kyiv). DNA from blood samples was extracted using the phenol-chloroform method and by the DNA-SORB-B (AmpliSense, Russia). The *BRCA1* mutation 5382insC was detected by multiplex mutagenically separated PCR using published specific primers [23]. Three primers were used to detect the mutation: one general, one specific to the mutation in question, and one specific to wild the type allele as described in Chan et al., 1999 [23]. PCR amplicons were analyzed by 8 % PAGE and 2 % agarose.

We screened a total of 193 breast cancer patients diagnosed ages 18 through 80 for the 5382insC mutation. A total of nine *BRCA1* 5382insC mutations were detected (4.7 %) (Table 1). Most carriers of the 5382insC mutation were younger that 40 years at the time of detection (ages 19, 27, 31, 34, 36, 38, 39), although two occurrences were found in older patients (52 and 44 years). This represents a frequency of occurrence of 7/79 (8.9 %) for individuals under age 40 and 2/114 (1.8 %) for patients older than 40. A mutation was found in 5 of 90 patients with a FH of cancer (5.5 %) and in 1 of 45 patients with no FH (2.2 %). 13 patients with a FH of cancer had precisely breast cancer in family (1 with mutation) and 77 patients with a FH had other cancer types: prostate, stomach, lung cancer and uterine fibroids (2 with mutation), also 14 patients had both, breast and other cancer types in FH, such as laryngeal, lung and prostate cancer (2 mutation carriers). The frequency of the 5382insC mutation is similar in patients with and without a FH of breast cancer (F = 0.01, *p* > 0.05).

**Table 1:** Frequency of the 5382insC mutation in breast cancer patients

Age	>30	30–39	40–49	50–59	60–69	≤70
n	24	54	48	34	27	6
Mutation	2	5	1	1	0	0
Frequency, %	8.3	9.2	2.0	2.9	0	0

In this study, we report that 4.7 % of unselected breast cancer patients from Ukraine carry a 5382insC mutation. This estimate is similar to those of previous studies that estimated the prevalence of the mutation between 2.5 % and 7.1 % (F = 0.45, *p* > 0.05) [14–18]. Combining the results of all studies, including ours, the pooled frequency for Ukrainian patients is estimated at 5.81 ± 0.9 % (38 cases out of the total 654 women patients screened).

Ukrainians are eastern Slavics by genetic background and are close relatives of Belarusians, Poles, and Russians. The estimate of the 5382insC mutation in the *BRCA1* gene for Ukraine (5.8 %) is higher than reported for Belarusians (2.5 %) (F = 9.23, *p* < 0.05) [24], but lower than that of Poles (10.4 %) (F = 12.21, *p* > 0.05) [25] and is similar to an estimate from Saint Petersburg, Russian (4.7 %) (F = 0.42, *p* > 0.05) [26].

The average age amounted 35 years in 5382insC carriers versus 45 years in non-carriers which is consistent with other reports indicating that the 5382insC mutation is a factor of genetic predisposition to breast cancer, which is also consistent with reports from other countries [27].

The 5382insC mutation of *BRCA1* is a frequent germline mutation in Ukrainian breast cancer patients. Interestingly, and in contrast to other reports, our results suggest no difference in 5382insC mutation frequencies between breast cancer patients with and without a FH of the disease. The presented data can confirm a noticeable contribution of *BRCA1* 5382insC mutation in breast cancer development in Ukraine and as there is no difference between the frequency of mutations in the groups with and without a FH it may justify to screen for 5382insC mutation all breast cancer patients totally, not primarily that who have a FH.

## Abbreviations

*BRCA1*: Breast cancer 1, early onset
FH: Family history
